# The Hospital Nursing Supplement

**Published:** 1895-09-14

**Authors:** 


"The Hospital" Nursing Mirror.

Being the Extra Nursing Supplement of "The Hospital" Newspaper.

[Contributions for this Supplement should be addressed to the Editor, The Hospital, 428, Strand, London, W.C., and should have the word "Nursing" plainly written in left-hand top corner of the envelope.]

### News from the Nursing World.

### LONDON HOSPITAL NURSING HOME.

It must be very satisfactory to all who are interested in the London Hospital to hear that the new wing which is to form a much-needed addition to the Nursing Home is to be begun forthwith. The present home may well be taken as a model of what such an institution should be, with its separate bed-rooms for each nurse and probationer, conveniently and tastefully furnished, its sitting-room and excellent library, and the hundred and one small comforts which Miss Lückes spares no pains in procuring for her nurses; but its powers of elasticity were long ago exhausted, and with the large private nursing staff to be accommodated more room is badly wanted. That the new home will be, if that is possible, even an improvement upon the older portion need not be doubted, for the Committee are evidently desirous of doing everything in their power for the comfort and well-being of the nursing staff.

### GUARDIANS AND DISTRICT NURSING.

A question has been raised as to the proper basis on which guardians should pay for the nursing of their outdoor poor, it having been suggested by some that the plan of payment by visit should be adopted instead of a fixed contribution to the District Nursing Association. In regard to this Miss Louisa Twining writes: "I can only say that the payment by visits is not adopted by any of the branches [of the District Nursing Association], and that a definite sum is contributed annually by an ever-increasing number of Boards of Guardians. I am quite aware that the Local Government Board sanctions the employment and entire payment of nurses for the outdoor poor alone; but, as the chief consideration was, as I understand, the reduction of the rates, this would involve an expenditure of £80 or £100 a year, instead of £20." There can be no doubt that it is best both for the guardians and the poor, especially for the poor, that the payment should be made in a lump sum rather than according to the number of visits made. Many a time will a nurse look in on a poor patient as she is passing, just to see that all is straight and right, when she would hesitate to do so if she knew that the charge for that visit would be marked up against that patient in the records of the relieving officer, and it is almost certain that, in the present condition of poor law administration, any outdoor case which was found to be running up a large bill for nursing would before long be offered the alternative of the house. No! better leave things as they are, where there is not sufficient work to justify the guardians in obtaining the exclusive services of a district nurse of their own, and let the outdoor sick and aged under the poor law feel that, in regard to their nurse at any rate, they are on the same footing as others in the village who are not yet paupers.

### A DESERVING SOCIETY.

The Hull District Nursing Association has a reputation for hard work and economical management which should secure for it ample support. A home for the nurses has now been secured, and a small debt which remains on the furnishing ought soon to be paid off by those acquainted with the excellent work of this society. Many incurable cases of cancer, phthisis, and paralysis have been skilfully tended in their homes, 4,691 visits being paid to patients in the past twelve months by Miss Furley, the lady superintendent, and her nurses. The latter are three in number, and there is plenty of work for all; and we trust that additional subscriptions will shortly remove from the committee all anxiety as to their future financial position.

### RURAL DIPHTHERIA.

Diphtheria is so irregularly distributed, both its virulence and its frequency varying greatly in different parts of the country, that there may still be some who hesitate to regard its increasing prevalence with the gravity which it demands. It is well, then, to draw attention to the dread with which it is regarded in those country districts where it prevails, and to point out that in the absence of proper means for its treatment and isolation by the sanitary authorities the sufferers are apt to be thrown on their own resources and to be isolated from all effective assistance in a manner which recalls the old description of epidemics in the middle ages. Only last week we read of an outbreak of diphtheria not far from Colchester and within fifty miles of London. Of three children living in a labourer's cottage two were attacked with diphtheria and both died. Then the third child was laid low with the same disease. In the meantime diphtheria showed itself among the four children next door, and one of them died. Such, however, was the dread caused by the outbreak that the undertaker, in bringing the three coffins, set them in the yard, leaving the parents to place the corpses in them and to screw them down. The coffins, as we gather from the *Halstead Times*, were then removed to the churchyard at Great Tey, where the graves had been dug, and the children were committed to the earth by their own fathers, the one assisting the other, and no burial service being performed. Since then two more children in the second cottage have died, and the father has been attacked by the disease. There is nothing very extraordinary in this little episode of village life. In districts where diphtheria is rife it doubtless could be matched, perhaps outdone, by many doctors or nurses of experience. Its interest lies in the illustration which it gives of the extraordinary virulence of the disease under some conditions, the helplessness of a rural population in the face of an outbreak, and the demoralisation caused by its ravages. We doubt if the true meaning of epidemic diphtheria is at all recognised or appreciated by many who try to throw cold water on the attempts made by science to perfect a means of cure for the disease. Those who know best are the first to hail the possibilities opened out by the discoveries of bacteriologists, not only in the direction of treatment but as offering a means by which a temporary immunity in the healthy may be induced which may protect them till the time of danger passes by.

### NIGHT NURSING IN IRISH WORKHOUSES.

It is difficult to understand why the Irish people so full of kindness and sympathy for each other, and in many ways so ready to make the most of the very wide ramifications of the poor law system of their country, should for so long have acquiesced in arrangements for the nursing of the sick inmates of their workhouses which cannot be described by any term milder than disgraceful. There is hope, however, of better things. In answer to a question in the House of Commons, Mr. G. Balfour stated that his attention had been directed to the numerous complaints regarding the ineffective and primitive arrangements for night nursing in many of the workhouse hospitals in Ireland, and that the Local Government Board had addressed a circular to each Board of Guardians throughout Ireland, calling their special attention to the night nursing, and requesting that the medical officer of each workhouse might be called upon to furnish a report on this most important matter, and that the Visiting Committee of each union, after consultation with the medical officer, should report as to any changes or improvements which might be carried out in the hospital. In speaking further on the subject, Mr. G. Balfour said that the defects referred to had been in every case ascertained and recorded, and he hoped they would be remedied without having recourse to the appointment of a commission, and the enforcement of its recommendations by more drastic measures. We feel sure that a little pressure from headquarters will have much influence for good in many dark spots in Irish poor law administration.

### THE TRURO DISTRICT NURSE.

The sick poor of Truro have for some time past had the benefit of the services of a nurse who has worked in connection with the Cornwall Trained Nurses' Home. This home, however, has recently been closed, and as a result it has become necessary to make some other arrangements for providing Truro with a district nurse. An influential committee have had the matter under their consideration for some weeks past, and a scheme has been devised whereby the good work may be continued, if a sum of from £70 to £80 can be provided annually. We can hardly believe that Truro will fail to provide this for so good an object.

### THE DROGHEDA WORKHOUSE.

The foundation-stone has been laid for the residence of the Sisters of Mercy, who are to be in future the nurses in connection with the infirmary of the Drogheda Workhouse. The Rev. J. Curry blessed the foundation-stone, and said that the event marked one of the greatest boons that ever happened to the poor and sick of Drogheda. It is much to be desired that it will prove so, for which purpose we hope the authorities will be careful to arrange that the Sisters of Mercy who undertake the duty be selected from those who have undergone a proper hospital or infirmary training.

### BALLYCASTLE WORKHOUSE.

At the meeting of the Board of Guardians at Ballycastle, Ireland, the medical officer of the workhouse reported concerning the nursing arrangements as follows: I beg to say that since the appointment of the present trained nurses the state of the infirmary generally has been much improved, the patients are kept clean, they are well attended to, and the medicines are carefully administered, but I am still of opinion that a grave defect exists in the nursing department in the want of a person with some knowledge of midwifery, and I regret that my suggestion that such a qualification should be made an essential in the appointment was not carried out. I am also still of opinion that an assistant above the position of a pauper should be appointed to act as night nurse, and to assist the nurse generally in the infirmary work, and who should have some knowledge of midwifery.

### LONG HOURS OF WORK.

Some correspondence has lately appeared in the Glasgow papers regarding the long hours for which hospital nurses have to work, it being stated that in some cases 14 hours a day is the rule rather than the exception. There can be no doubt that no institution should ever require a nurse to remain on duty so long as 14 hours except in a case of emergency; and we believe that in most of the better-managed hospitals in this country the hours of work are now much shorter than they used to be. It should, however, be mentioned that a nurse's hours of duty cannot be fairly estimated by taking the times of coming on duty in the morning and leaving off in the evening. It often happens that the stress of the work falls at the two ends of the day, and thus that although a nurse may come on duty at seven a.m. and not finally leave off till between eight and nine p.m. she may have had a certain amount of time in the course of the day entirely off duty and completely at her own disposal. Nevertheless we find it still remains true that in many hospitals the hours of duty are much longer than they should be.

### ABERDEEN DISTRICT NURSING ASSOCIATION.

A bazaar is about to be held in Aberdeen with the object of raising money for providing an enlarged home for the nurses working in connection with this association, which during the three years of its existence has been doing much good. The association is affiliated with the Queen Victoria Jubilee Institute for Nurses. Miss Armstrong entered single-handed upon the work in 1892, but since that time additional nurses have been associated with her, and recently the trustees of the Chalmers' Fund purchased a large and conveniently situated house on the Castle Hill with the view of its being made the Nurses' Home. By this welcome gift one of the greatest difficulties of the association—that of providing a suitable residence for the nurses—has been removed, and the way has been prepared for a large extension of the work. Funds, however, are required, not only to meet the annual expenditure, but for furnishing, and to this end the bazaar has been organised.

### Elementary Physiology for Nurses.

By C. F. Marshall, M.D., B.Sc., F.R.C.S.

### VI.—THE CIRCULATORY SYSTEM—(*Concluded*).

### Tonic Contraction of Arteries.—The Sympathetic Nerves.

Variations in the size of the arteries are exceedingly important, for they are the means by which the blood supply to any part is regulated. The quantity of blood is constant, yet at one time the brain may want more, at another time the stomach, &c. The small arteries have muscular walls, and are naturally slightly constricted. This "tonic contraction," as it is called, is liable to relaxation or "inhibition" through the nervous system. This leads to increased size of the artery and increased supply of blood to the part to which the artery goes. This change is effected very quickly, and a familiar illustration of it is the act of blushing, when a sudden reddening of the surface occurs owing to the dilation of the small arteries through the action of the nervous system.

The nerves which govern the arteries are called the sympathetic nerves; they have other functions as well as that governing the arterial system. When the sympathetic nerve in a rabbit's neck is cut the ear flushes at once, owing to the restraining or inhibitory action of the nerve on the small arteries being removed, the vessels becoming increased in size and allowing more blood to pass through them.

Inflammation is a somewhat analogous to the above, and is characterised by the increased supply of blood to a particular part. The chief signs of inflammation are redness, swelling, heat, and pain. The phenomena of inflammation may be studied in the web of the frog's foot by applying some irritant such as mustard. First the arteries dilate and the stream of blood flows quicker; secondly, the stream slackens while the arteries are still dilated; this stage is known as stasis, during which the small veins, arteries, and capillaries become choked with corpuscles. After this occurs migration of the white and some of the red corpuscles through the walls of the capillaries. Finally, when the irritating substance is removed, there is re-establishment of the circulation.

### The Rate of Flow of Blood.

This is very different in different vessels, and depends on their size. It is clear that, roughly speaking, the same amount of blood must return by the veins as is sent out along the arteries. The veins are much larger than the arteries, and therefore the flow of blood is slower in them. In the large arteries the rate is twenty inches per second; in the large veins eight inches. At each division of the arteries the area gets larger, and thence the flow of blood slower.

The capillaries, though individually very minute, are yet so numerous that their combined area in any part is greatly in excess of that of the arteries supplying it. Hence the rate of flow is very slow in the capillaries, only about one-hundredth inch in a second, or an inch in about a minute and a-half. As a complete round of circulation takes only half a minute and involves two sets of capillaries, it is clear that the capillaries in any part of the body must be very short. We may compare the flow of blood to the flow of a stream of water, which is swift where the bed is narrow; slower as it widens out; hardly perceptible as the stream enters a lake, but swifter again on leaving it.

The pulse is the sudden swelling of an artery due to the heart-beats, for the intervals between successive beats of the pulse and the heart are the same. Yet they are not simultaneous, the pulse coming distinctly after the heart-beat. The pulse is, in fact, a wave of blood passing from the heart along the arteries, and due to the elastic walls of the arteries. The further away from the heart, the longer is the interval between the heart-beat and the pulse.

### Blood-pressure.

The arteries are not rigid tubes, but are highly elastic, like indiarubber. Hence, on each contraction of the ventricle, driving a certain quantity of fresh blood into the aorta, room is found for this blood by the expansion of the artery rather than by the whole stream of blood being forced along bodily. Directly the ventricle ceases to contract this pressure is removed and the aorta contracts, owing to its elasticity. It behaves, in fact, very much as an indiarubber tube would do. This contraction causes dilatation of the artery further along, which is thus passed along the artery as a wave, and causes the pulse.

In the veins and capillaries there is no pulse, for since the combined area of the arteries increases at each division, and since the combined area of the capillaries is much greater than that of the arteries, the pulse must be rapidly diminished in force, and from this cause alone might become imperceptible in the capillaries. The capillaries, being very small, offer much resistance to the passage of blood owing to friction. The arteries, therefore, have not time to empty themselves before the next heart-beat comes, and consequently they are always over full. The walls of the arteries, being elastic, tend to contract, and so cause the pressure of blood to be constant instead of intermittent, which it would otherwise be if the arteries were rigid tubes instead of being elastic. Therefore there is no pulse in the veins, except when the heart beats unusually feebly.

The lymphatics are a second set of vessels which occur in all parts of the body, commencing in the interstices between the component parts. They serve to return to the blood nutritious matters exuded, but not used. They also play an important part in the absorption of food, as we shall see in a future lecture. The lymphatics consist of thin walled vessels of more irregular shape than the capillaries or veins. They open chiefly into a large terminal vessel, the thoracic duct, which joins the venous system at the left side of the root of the neck, close to the jugular vein.

### Destruction of Red Corpuscles.

As the duration of a red corpuscle of the blood is supposed to be only three or four weeks they must be continually being destroyed. This destruction goes on probably in the liver, spleen, and the bones; at any rate, the colouring matter—hæmoglobin—is excreted by the liver and forms a constituent of the bile.

### Manufacture of Blood Corpuscles.

This must be always actively going on, and the red marrow of the bones and the spleen appear to be the chief manufacturing centres of the corpuscles of the blood.

### Novelties for Nurses.

### THE NON-RUN-AWAY BANDAGE.

Messrs. Reynolds and Branson, of Leeds, whose novel conveniences for tending the sick we have constantly the pleasant task of bringing before our readers, are again introducing a most useful contrivance. At the suggestion of Dr. A. Duke, of Cheltenham, they have obviated the liability of a bandage slipping and unrolling when being used. This is done by a piece of round elastic being passed through the middle of the roll of bandage, and holding a small cardboard roller against the outside of the bandage, so that although, in unrolling, the bandage is easily released by the pull upon it, yet, so long as no traction is exercised upon it, the roll is held firmly together. The cost is slight, the convenience is great, more especially when, in the absence of doctor or nurse, an individual has to tend his own injury. A sample box of one dozen can be obtained from Messrs. Reynolds and Branson, Briggate, Leeds.

### French Schools for Trained Nurses: Their Origin and Organisation.

By Madame W. Vignal.

### V.—THE BICETRE AND PITIÉ SCHOOLS.

The hydropathic department at the Bicêtre Hospital is admirably arranged. In the section where the idiot children are treated there is a spacious bath-room for their exclusive use. Dr. Bourneville, with his usual energetic good sense, insisted that the nurses, both male and female, should be taught how to administer the douche and other baths, and also how to perform epilation.

At first sight these duties may appear to be easy of performance, but in reality both the knowledge and practice necessary are wanting, and in the majority of the Paris hospitals both shower and other baths are badly given. All nurses, both male and female, ought to learn this part of their profession; accidents would thus be prevented, and the patients would not be exposed to the dangers to which the ignorance of the bathing-nurses render them liable. In order to minimise the ignorance of the Bicêtre Hospital staff in relation to this important subject, M. Bourneville directed that several dictations in relation to it should be given to the pupils of the Bicêtre and Salpêtriere schools. The learned director of the nursing schools is fully alive to the importance of this detail in the education of the pupils. In an address given last year he said: "If the Pitié Hospital School becomes a higher school (*une ecole de perfectionnement*) good practical lessons on the art of giving baths of every kind must be organised; moreover the bathing department should be entrusted only to the male and female nurses who have a thorough practical knowledge of the processes acquired under the superintendence of competent people. Such a lamentable fact as that of a stone-breaker being taken without any preparatory instruction as a hospital 'doucher' would then be only true of the past, and not possible of recurrence in the present or future." In the same address Dr. Bourneville treats the question of epilation as follows: "Since the opening of the Bicêtre Hospital in 1856 there have always been a certain number of children suffering from ringworm. In the idiot children's section, from 1879 to 1894, notwithstanding the strictest supervision and the utmost care, there have always been some cases. Every year child patients are admitted with this affection, and, notwithstanding every possible precaution, contagion, facilitated by the mental condition of the children, unavoidably occurs. The number of children suffering from ringworm, owing to the skill and zeal of the female nurses entrusted with their care, becomes less and less; nevertheless, the average is sufficiently important to render it necessary that male and female nurses should know exactly how to treat this affection, and epilation is one of the expedients. To learn how it is practised is necessary, but the system of keeping the nurse so long in the same hospital deprives them of the opportunity of acquiring this knowledge, and the greater number of pupils will leave these schools without doing so. At Berck the same thing has occurred." A considerable number of children in the Berck Hospital were attacked with ringworm, and a female nurse having a special knowledge of epilation was sent there from the St. Louis Hospital in Paris.

Before leaving the Bicêtre School it will not be out of place to glance at the material condition of the male and female nurses in the different Paris hospitals; how they are clothed and fed. Their food is now much better than it was formerly. In most of the hospitals the management has introduced a little variety in the dishes composing the daily meals. It is to be hoped that this improvement will become general, and that in all hospitals and State charitable institutions the nurses, male and female, will be provided with suitable food. Formerly they were not furnished with salt and mustard, and had only one plate each which served for the different kinds of food. Some hospital directors, with a desire to introduce a little cleanliness and comfort into the domestic arrangements of their nursing staff, have ordered the new sheets to be used as tablecloths. This arrangement, rough as it is, would be an improvement in many refectories of the Paris hospital nursing staff, where the tablecloths and finger napkins are as a rule far from clean. The clothing of the male nurses, which is made of thick cloth, is excellent for winter wear, but in the intense heat of Paris summers it must entail much needless suffering and fatigue on the wearers.

As we have already stated, the School of the Pitié Hospital is one of "*perfectionnement*" where the pupils perfect themselves in the knowledge exacted by their calling. There is no primary section in this school, which has now entered its thirteenth year of existence, and is the last we shall have to describe. The number of pupils entering this school increases every year. In 1890-91 there were 345 pupils; in 1891-92, 528; 1892-93, 530; 1893-94, 529. Among those pupils 98 were nurses belonging to the Pitié Hospital, 346 came from hospitals and asylums, 84 were pupils unattached to any hospital or asylum.

From 1892-93 the Pitié sent 119 pupils to its school; other hospitals and asylums, 306; unattached pupils numbered 350. From 1893-94 the pupils from the Pitié Hospital numbered 86; from other hospitals and asylums, 306; the number of unattached pupils was 105; total, 497. In 1893-94 the Pitié sent 86 pupils, other hospitals and asylums, 399; the unattached pupils were 44, the total being 529.

The number of pupils at this school has increased to such an extent that the lecture theatre is too small to accommodate them. Overcrowding is inimical to comfort, and has prevented a number of pupils from attending the lectures, and thus partially explains the absence of many students in the course of the year.

The oral examinations are in general very satisfactory; the answers are prompt and correct. The bandaging, however, is far from good, especially that done by the male nurses.

Both male and female nurses ought to be called upon to perfect the senses of sight and smell, in order to recognise quickly and correctly the different drugs and remedies, and thus avoid making mistakes. Manual dexterity is equally or even still more important. The bandages placed to prevent dressings from slipping should be quickly and dexterously placed *in situ*. In order to attain this perfection, the nurses must practise on a good-natured fellow-student or a willing patient.

(*To be continued.*)

### Appointments.

[It is requested that successful candidates will send a copy of their applications and testimonials, with date of election, to THE EDITOR, The Lodge, Porchester Square, W.]

Evesham Cottage Hospital.—Miss Radford has been appointed Matron of this hospital. She was trained at the Ancoats Hospital Manchester, where she subsequently became sister, which post she leaves for the present one.

### Minor Appointments.

Rural District Council's Hospital, East Ashford.—Miss Beatrice Meredith has been appointed charge nurse of the above hospital. She was trained at Addenbrooke's Hospital, Cambridge, and subsequently held appointments at the Leicester Fever Hospital and the Leeds Borough Hospital.

to that of a general hospital, each ward having a nurse in charge (both day and night), one trained first assistant, and one or two untrained nurses (according to the number of patients), who, instead of being called probationers, are styled second assistant nurses. With regard to the first sentence, viz., "The board being reticent as to all its nursing arrangements," we feel quite certain that visitors would be most cordially welcomed at any time, and conducted over the hospitals.

[The question which we raised was as to the status of these untrained persons who, on being engaged, become second-class assistant nurses. If they are merely probationers, as is suggested in one of the above letters, it would be better to call them by their proper name, and engage them for a proper period, and thus, on the one hand, prevent them afterwards posing as nurses, and, on the other, make it impossible for the public to think that the Asylums Board nurses (be they second-class assistant or any other grade) are untrained.—Ed. *T. H.*]

### St. Olave's Union Infirmary.

A paragraph in the *Star* of September 5th having been brought to our notice reflecting upon the Matron of the above infirmary, we have carefully investigated the facts, which appear to be as follows. Hearing that the Guardians were contemplating the appointment as probationer of a servant in the employ of the steward (of whom Miss Evans, the matron, strongly disapproved, for very sufficient reasons from a nursing point of view, as a candidate) the following correspondence has taken place:—

To the Chairman and Members of St. Olave's Board of Guardians.

Gentlemen,—I am sorry that I did not know that M. H., one of the servants of this institution, had applied to the committee for the post of probationer nurse. In my opinion she is a very unsuitable candidate. I have known her for the past three years, and have not found her very satisfactory; and the fact that she has been a servant here will render it injudicious for her to be promoted in this institution. This I explained to her some weeks ago, and told her it would be wiser of her to apply elsewhere. I shall, therefore, feel glad if the Board will not confirm her appointment, as it will not add to the harmony of the nursing department or to the welfare of this institution.—I am, &c.,

Marion M. Evans, Matron.

Madam,—I am directed by the Guardians to acknowledge the receipt of your letter of the 29th inst., addressed to the chairman and members of the Board of Guardians with reference to the recommendation of the Visiting Committee of the infirmary to appoint M. H. as a probationer nurse at the infirmary.

In reply, I am directed, firstly, to express to you the strong disapproval of the Guardians at the course you have seen fit to take in respect of the recommendation in question; secondly, that in their opinion your action in writing a letter framed in such terms and expressing such opinions was most injudicious and unwarranted; thirdly, I am to point out to you that the appointment of the officers and servants of the infirmary is a question entirely in the hands of the Guardians, who cannot allow their decisions and recommendations in regard thereto to be questioned in any way by you; fourthly, that it is entirely outside your province to dictate to the Guardians, but to obey their orders and carry out their instructions; fifthly, that should you address the Board in similar terms on any future occasion, they will have to consider your position at the infirmary and the terms and conditions of your appointment.—I am, &c., E. Pitts Fenton, Clerk.

The correspondence speaks for itself, and needs no comment. We may, however, add that Miss Evans has worked harmoniously for five years at the head of the nursing department of St. Olave's Infirmary, cordially supported by the medical officers on all occasions, and that the majority of her nurses are devoted to her, having, as one of them has stated, "always found her just and considerate to them." She has successfully carried through many reforms, and that her department is well organised and administered a visit to the infirmary wards and nurses' home will amply prove.

### "The Ockley System."

We are not responsible for the phrase "The Ockley System," as describing the method of providing village nurses adopted by the Ockley Nursing Association. It is taken from a letter which appears in the *Parish Councillor* in defence of this system, and we adopt it because it seems well to have some short definition of this plan for providing attendance on the sick by a class of women who clearly are not entitled to be called nurses.

The writer of this letter says: "It appears to me . . . . that three months' training—*i.e.*, six weeks in a maternity hospital and six weeks in an ordinary hospital—should be and is sufficient to enable a woman to look after ordinary maternity cases and illnesses." We, on the other hand, writing with full knowledge and responsibility, repeat that a nurse cannot be made in three months, much less both a nurse and a midwife, and those who understand the subject will agree that it is a misnomer to give to women who have only had such a training the title of "trained nurse." It is stated that the nurses under the Ockley system "are not for severe cases, but for ordinary cases of illness, which are treated in the cottage home, and for maternity cases, which, as a rule, require only ordinary care and knowledge." Now, in reference to this system, it is well to speak with certain voice, for it is admitted even by its supporters that "the more training the better," and that the excuse for the "Ockley system" is a mere matter of economy, and we say that such a system is not only opposed to the opinion of those who have had the most practical experience in the education of nurses, but is in effect setting up a standard of training for the village nurse far lower than that which is made obligatory by the Local Government Board for the nursing of out-door paupers.

Before issuing the general order enabling boards of guardians to employ district nurses for their out-door sick, the subject of the sort of person to be so employed was carefully considered by the Local Government Board, and in a circular letter addressed by them to boards of guardians, the following paragraph appears:—

The Board considers that it is of great importance that the persons who may be appointed by guardians to the office of district nurse should have had thorough practical training in the nursing of the sick; and Article III., therefore, directs that no person shall be appointed to the office who has not undergone, for one year at least, a course of instruction in the medical and surgical wards of a hospital or infirmary, being a training school for nurses, and maintaining a resident physician or house surgeon. A longer period of training than one year would seem desirable, although the Board have not deemed it expedient to insist upon it as an indispensable condition.

Now this is the only official definition of a trained nurse which we know of; it is the standard imposed on boards of guardians, who have never been famed for any excess of zeal in providing better nursing than is actually necessary, and it defines the sort of nursing fitted for the poorest of the poor, who, in popular but we think very unseemly language, are described as 'tainted' with the 'stain' of pauperism. If for them, notwithstanding the fact that all the severe cases would usually be taken into the infirmary, such a standard is required, what are we to think of a system according to which the sick poor in villages—the deserving poor, if we may use the phrase—who have managed to support themselves without asking for help from the poor law, are to be nursed by women who have only undergone a six weeks' training in an ordinary hospital? If such nursing is given free, it is, we fear, but another instance of cheap charity; if, on the other hand, it is provided by the villagers for themselves on co-operative principles, these poor people ought to know how different is the article they get from that which is considered essential for the much-despised pauper.

Another matter also should be brought to the notice of those responsible for the introduction of this system of combined village nurse and midwife, viz., that the Local Government Board, for reasons obvious enough, definitely state in Article V. that "no district nurse shall undertake the duties of a midwife."

We commend these points to the careful consideration of those who, with the best of motives, no doubt, are endeavouring to introduce a system of so-called district nursing which is likely to lead to many evils.

### The Book World for Women and Nurses.

[We invite Correspondence, Criticism, Enquiries, and Notes on Books likely to interest Women and Nurses. Address, Editor, The Hospital (Nurses' Book World), 428, Strand, W.C.]

The Bearer's Companion: First Aid to the Injured and Management of the Sick. An Ambulance Handbook, and Elementary Manual of Nursing for Volunteer Bearers and Others. By E. J. Lawless, M.D., D.P.H., Surgeon-Major 4th V.B. Surrey Regiment. With forty-nine engravings. (Edinburgh and London: Young J. Pentland. 1894.)

The author tells us he has endeavoured to arrange "First Aid Instruction" to suit those attending regimental classes, because the "Medical Staff Corps Manual" is scarcely adapted to the more limited needs of a regimental bearer section, or of a volunteer brigade bearer company. We should not regard this book as adapted to "limited needs," for scarcely anything appertaining to the subject is omitted from its pages. Indeed, the book is almost too professional for average members of volunteer and civil ambulance classes. The course of the principal arteries is given clearly, and also the means for arresting "bleeding," although somewhat too technical. There is an excellent lesson on bandages, illustrated by woodcuts; fractures and splints are similarly illustrated. Lesson eight contains advice on wounds, and on first dressings for burns, scalds, injuries of the eyes, stings, and bleeding from the nose. For the latter lying on the back is suggested. In such circumstances an individual certainly should not sit up and bend his throat over his collar and hang down his head. But as nose-bleeding, if severe, is usually arterial, sitting up with the head thrown back will bring gravity into play, and thus tend to prevent blood being pumped upwards, acting in much the same manner as extending the arms "at full length above the head," advocated by the author. For the treatment of the apparently drowned, Sylvester's, Marshall's, Hall's, and Howard's methods are given; but we are not told which is the best to adopt. There are long lessons on "stretcher carrying" and "hand seat drill." On the march with stretchers the "broken step" is advocated, as causing less motion of the stretcher. The loading and unloading of ambulance waggons concludes Part I. An appendix of questions and answers on the foregoing part is attached. As demonstrating the information the author expects his readers to possess, we quote a few of the questions: "Name the bones forming the cavity of the skull." "Mention those forming the face, and the important parts they enclose." "Name the bones of the pelvis." "What are the uses of the periosteum?" "How are the lungs concerned in the circulation of the blood?" The second part of Dr. Lawless's book is headed "Management of the Sick," and is divided into seven chapters, which contain excellent advice but too much repetition, the same instructions being given in the second part of the book as in the first on some subjects. In an appendix to Part II. "fresh air and ventilation" are discussed, also diphtheria, typhoid, small-pox, erysipelas, &c. There can be no doubt that it is an excellent work, although more adapted for the advanced than for the ordinary student of "First Aid to the Injured." The value of the work is enhanced by a copious index, and it is got up most creditably.

MAGAZINES OF THE MONTH.

The Cornhill Magazine for this month contains nothing of any very special interest, but a uniformity of excellence pervades the writing throughout the pages. The two stories, "Cleg Kelly," by S. R. Crockett, and "The Sowers," by Henry Seton Merriman, continue in serial form. There are two short tales complete in this number, which are worth reading, and two pleasant papers, one about "Our Stone Crusaders," and the other "About Amber."

The Sunday at Home has some good reading, and is improving in its illustrations. Here is a reproduction of Mr. Hemy's picture, "Where Two or Three are Gathered Together in My Name," and a good article accompanying it about "Sunday in the North Sea."

Cassell's Family Magazine.—Among the most attractive articles this month is Dr. A. H. Japp's chapter on "Animals as Beggars"; it is charmingly interspersed with studies of animals illustrating the writer's remarks. There are one or two good short stories, and a paper on "The Fourth Estate in London," by A. F. Robbins, introduces those not already initiated into the ways of journalism in the metropolis.

### "The Hospital" Convalescent Fund.

The hon. secretaries beg to acknowledge with thanks 2s. 6d. from "A Nurse who has Enjoyed her Holiday." The above contribution displays a most kindly thought, which much enhances its value. The nurse in question probably was in the enjoyment of health during her holiday, and her kind sympathies were therefore especially enlisted for those less fortunate sisters who, without the blessing of good health, are still unable to procure the needed holiday for themselves.

### Notes and Queries.

The contents of the Editor's Letter-box have now reached such unwieldy proportions that it has become necessary to establish a hard and fast rule regarding Answers to Correspondents. In future, all questions requiring replies will continue to be answered in this column without any fee. If an answer is required by letter, a fee of half-a-crown must be enclosed with the note containing the enquiry. We are always pleased to help our numerous correspondents to the fullest extent, and we can trust them to sympathise in the overwhelming amount of writing which makes the new rules a necessity. Every communication must be accompanied by the writer's name and address, otherwise it will receive no attention.

### Queries.

(245) *Medical Homes.*—Can anyone tell me of a town in which there is an opening for a private medical and surgical home and a staff of private nurses?—*Inquirer.*

(246) *Doctor's Degree.*—(1) Can you tell me the approximate cost of a nurse obtaining a doctor's degree? and (2) what book contains all the medicines in the British Pharmacopeia, with their uses?—*H. M.*

(247) *L.O.S.*—(1) What are the dates for the remaining part of this year for the examination for the qualification "Licentiate of the Obstetrical Society of London," for midwives? (2) What text-book would you recommend to read up for the examination? (3) Where could I procure a set of recent examination questions?—*Toby.*

(248) *Bicycle.*—Can you help me to procure a suitable bicycle?—*District Nurse.*

### Answers.

(245) *Medical Home* (*Inquirer*).—We publish your query hoping that someone acquainted with a locality such as you are seeking may answer it. We take this opportunity to warn you earnestly that the establishment of a medical home, unless directly under medical patronage, is a most hazardous undertaking. We learn that quite a third of the applicants for assistance from the Royal National Pension Fund Benevolent Fund come from nurses who have failed in these enterprises.

(246) *Doctor's Degree* (*H. M.*)—(1) The cost of a medical education where no drawbacks occur is estimated to amount to £700. (2) A good book on Materia Medica will give you the information you require.

(247) *L.O.S.* (*Toby*).—(1 and 3) Write to the Secretary of the L.O.S, 54, Berners Street, W. (2) "First Lines of Midwifery," by Herman (Cassell and Co.), is a small and useful book; and Haultain's "Practical Handbook of Midwifery" (*Scientific Press*, 428, Strand) is a complete and excellent work on the subject.

(248) *Bicycle* (*District Nurse*).—You had better go and inspect for yourself a number of bicycles at a warehouse where they keep most kinds, such as Goy's, in Praed Street, W., and explain the kind of machine you require. Numbers of bicycles are to be had in the autumn very little used, and you might watch the columns of the "Bazaar and Mart." Don't buy without advice.

*Iota.*—This inquirer and others are reminded of the regulation we have made not to reply to queries unaccompanied by name and address.

### Everybody's Opinion.

[Correspondence on all subjects is invited, but we cannot in any way be responsible for the opinions expressed by our correspondents. No communications can be entertained if the name and address of the correspondent is not given, or unless one side of the paper only be written on.]

### HOME FOR NURSES.

S. A. writes: When nurses have made a connection, and are tired of living in a box, they generally take a room for themselves, in order to have a "home." It is charming to have one's books and pictures about one, but the charm has drawbacks—such as returning from a three months' case and finding one's bed damp; or coming home so tired that one foot will hardly step before the other, and yet no supper till one goes out and buys it. My idea is to build a house, with about 150 bed-rooms, a large drawing-room, and dining-room. The bed-rooms, unfurnished, to cost from 6s. to 7s. a week. Food to be paid for only when at home. I will not take up your valuable space with details, but would ask nurses who think such a home likely to suit them to write to me at 14, Queen-square, W.C. Of course, it depends upon members whether such a residential club could be made to pay.

[Whilst agreeing with S. A. as to the desirability of such a refuge for nurses, we would warn her that unless she has ample capital she would find herself in serious difficulties before returns began to come in. We consider 6s. to 7s. a week a heavy payment for an unfurnished room unused during a large portion of the year, and though many nurses might like the advantages offered whilst out of work, they would not care to pay one-fifth of their earnings in rent. Financially, the return, if any, which S. A. could expect would be very small.—Ed. *T. H.*]

### CLUB ROOMS FOR NURSES.

A representative member of the medical profession writes to us as follows: All who are interested in nurses, and all who wish to see the benefit of good nursing placed within the reach of people of all ranks, should set their faces against a project which has been set on foot by the Royal British Nurses' Association. The proposal is that club rooms should be established for the use of members of the association in all the towns of the United Kingdom; and it is suggested that as clergymen's daughters are often busily occupied in their fathers' parishes, so doctors' daughters might find congenial work in organising and serving as honorary secretaries to local nursing centres such as the Royal British Nurses' Association wishes to set up. The whole affair is specious and ingenious; there is a distant flavour of Royalty about it; and there are plenty of doctors' daughters who will like to fuss among the nurses, especially as they are to be in touch with what one paper gushingly speaks of as "the headquarters of the nursing army," meaning thereby the offices of this association. It behoves us, therefore, to warn those who may be tempted to devote time and money to such schemes that they are but being made use of by the managers of one particular section of nurses for the benefit and advertisement of their own particular association. The Royal British Nurses' Association is a body which has entirely failed to draw to itself the mass of the trained nurses in this country; it in no way represents the nursing profession. At its meetings harmony has so little prevailed that its Royal President, H.R.H. Princess Christian, lately declined to attend unless assurances were given that they would be more properly conducted; and it is quite certain that the institution of clubs up and down the country for the exclusive use of members of this association will be a means of introducing into the daily life of many nurses the sort of feuds which have hitherto been left to their central committee. If looked upon as a means of obtaining a good advertisement, free gratis and for nothing, all at the expense of some innocent "doctors' daughters," for the benefit of a commercial institution, it is no doubt a clever move; but it will do great harm to the nursing profession, and will introduce discord wherever it succeeds. The membership of the Royal British Nurses' Association is, after all, a matter of payment, and so far from this body being representative of trained nursing, the cleverest nurse among them would cease to be a member if, seeing she got no good from it, she declined to send her subscription. She would, however, by no means become any worse a nurse thereby, and thus of course there are leakages in the subscription list. We do not wonder then at the committee casting about for some new attraction to reconcile their members to the yearly tax, but we do protest against the public being asked to subscribe for, and "doctors' daughters" to organize, these nurses' clubs as if they were to benefit nurses at large, when really the tea and muffins so provided are merely intended as a bait to draw subscriptions from members of the British Nurses' Association who otherwise, so little is its use or attractiveness, would throw up all connection with it.

### HOW SMALL-POX PATIENTS ARE NURSED.

Sir,—In your issue of the 7th inst. there appears an annotation entitled, "How Small-pox Patients are Nursed," in which you make some adverse comments on an advertisement which was inserted in a daily paper inviting applications for the post of second-class assistant nurses at this hospital. I trust you will find room for a reply to your criticisms. Natural inferences for a person reading your annotation to make are: (1) That the nurses employed in nursing small-pox at this hospital are untrained; (2) that this hospital is peculiar in the constitution of its nursing staff; and (3) that for the nursing staff of such a hospital to be so constituted is, to use your expression, "utterly indefensible." (1) From the wording of an advertisement for second-class assistant nurses, it is an extraordinary deduction to make, as you appear to make, that it is considered that there is "no training necessary for the nursing of small-pox." A perusal of the advertisement columns in recent numbers of The Hospital would have shown you that this is far from being the case, and that the nursing staff of this hospital (excluding superintendents of nurses) is constituted as follows: (*a*) Charge Nurses, who must be able to produce a certificate showing three years' training in a recognised training school for nurses. (*b*) First-class Assistant Nurses, who must be similarly qualified by one year's training. (*c*) Second-class Assistant Nurses, in whose case no training (in the above sense) is necessary (but who are not necessarily inexperienced). (2) A reference to your advertisement columns would also have shown you that the constitution of the nursing staff of this hospital is identical with that of the fever hospital of the Metropolitan Asylums Board. (3) The appointment of untrained women to the lowest grade of the nursing staff occurs, I suppose, in every general hospital in London. I therefore fail to see why such a proceeding, usual in the case of every other disease, is "utterly indefensible" in the case of small-pox. There should be much virtue in a name if the substitution of the term "Second-class assistant nurse" for that of "Probationer" can make such a vital difference.—I am, &c.,

J. F. Ricketts,
Medical Superintendent of the Small-pox Hospital Ships.

Hospital Ships, Dartford, September 10th, 1895.

"Two other Correspondents" write: In answer to the paragraph in the last week's issue of "The Hospital Nursing Mirror," headed "How Small-pox Patients are Nursed," we should like to inform you that the nursing arrangement at the Metropolitan Asylums Board hospitals is conducted similar

